# Impact of comorbidity on the outcome in men with advanced prostate cancer treated with docetaxel

**DOI:** 10.1515/raon-2015-0038

**Published:** 2015-11-27

**Authors:** Andrej Zist, Eitan Amir, Alberto F. Ocana, Bostjan Seruga

**Affiliations:** 1 Department of Medical Oncology, Institute of Oncology Ljubljana, Ljubljana, Slovenia; 2 Division of Medical Oncology and Haematology, Princess Margaret Cancer Centre, Toronto, Canada; 3 Medical Oncology Department and Translational Research Unit, Albacete University Hospital, Spain

**Keywords:** metastatic castration-resistant prostate cancer, comorbidity, chemotherapy

## Abstract

**Background:**

Men with metastatic castrate-resistant prostate cancer (mCRPC) may not receive docetaxel in everyday clinical practice due to comorbidities. Here we explore the impact of comorbidity on outcome in men with mCRPC treated with docetaxel in a population-based outcome study.

**Methods:**

Men with mCRPC treated with docetaxel at the Institute of Oncology Ljubljana between 2005 and 2012 were eligible. Comorbidity was assessed by the age-adjusted Charlson comorbidity index (aa-CCI) and adult comorbidity evaluation (ACE-27) index. Hospital admissions due to the toxicity and deaths during treatment with docetaxel were used as a measure of tolerability. Association between comorbidity and overall survival (OS) was tested using the Cox proportional hazards analysis.

**Results:**

Two hundred and eight men were treated with docetaxel. No, mild, moderate and severe comorbidity was present in 2%, 32%, 53% and 13% using aa-CCI and in 27%, 35%, 29% and 8% when assessed by ACE-27. A substantial dose reduction of docetaxel occurred more often in men with moderate or severe comorbidity as compared to those with no or mild comorbidity. At all comorbidity levels about one-third of men required hospitalization or died during treatment with docetaxel. In univariate analysis a higher level of comorbidity was not associated with worse OS (aa-CCI HR 0.99; [95% CI 0.87–1.13], p = 0.93; ACE-27: HR 0.96; [95% CI 0.79–1.17], p = 0.69).

**Conclusions:**

Men with mCRPC, who have comorbidities may benefit from treatment with docetaxel.

## Introduction

Men with advanced prostate cancer are usually treated with hormonal therapy, which increases the risk for the development of comorbid conditions, such as diabetes, osteoporosis and cardiovascular disease.^1^–^3^ Most men with advanced prostate cancer receive hormonal therapy for several years and the average patient with metastatic castrate-resistant prostate cancer (mCRPC) in every-day clinical practice is 70 years old.^4^ Observational studies show that at this age more than 75% of cancer patients have at least one comorbid condition, with more than 30% having moderate or severe comorbidity.^5^

Based on improved overall survival (OS) in the TAX 327 and SWOG 9916 trials, docetaxel once every 3-weeks (hereafter Dq3w) in combination with prednisone is the standard treatment for men with mCRPC.^6^,^7^ A post-hoc analysis of the TAX327 showed that tolerability and efficacy of Dq3w appear less favourable with advanced age.^8^ Men with mCRPC who are treated with docetaxel in everyday practice may be less selected, older and have more comorbidities as compared to those treated with docetaxel in the pivotal randomized clinical trials.^4^,^9^ Outside of clinical trials, about 20–40% of men with mCRPC never receive treatment with docetaxel.^10^ Presence of comorbid conditions and/or poor performance status may be the reasons that these patients are not treated with docetaxel.^11^–^15^ Although comorbidities are known negative prognostic factors for OS in men with early prostate cancer their prognostic role in men with mCRPC is less clear.^11^–^13^ We hypothesized that comorbidity has detrimental effect on the outcome of men with mCRPC who are treated with docetaxel. Due to the dose reductions, docetaxel may be less effective in comorbid men. Furthermore, treatment with docetaxel may increase the risk for toxic deaths in men with mCRPC. Here we explored the impact of comorbidity on the efficacy and tolerability in men with mCRPC, who were treated with docetaxel in a population-based observational study.

## Patients and methods

### Study population and data collection

In this population-based observational study we included men with mCRPC who were treated with docetaxel and subsequent systemic therapies at the Institute of Oncology Ljubljana between January 1, 2005 and June 27, 2012. Men with mCRPC, who receive docetaxel in routine clinical practice, have usually performance status (PS) 0–2 and therefore PS was not evaluated retrospectively in this study. Optimal dose intensity of docetaxel was 25 mg/m^2^/week, which served as a denominator in the calculation of the relative dose intensity (RDI).

An optimal comorbidity index for prostate cancer patients is not established.^16^ We used the age-adjusted Charlson comorbidity index (aa-CCI) which is a composite index of 19 conditions weighted from 1 to 6 points and adjusted for age with each decade above 50 years of age counting for an extra point with a total score of 0–35 points. Point scores can then be classified into prognostic categories.^17^ Categories were formed using the same cut-off values as in the original publication and, for easier differentiation between them, tagged as “none” (0 points), “mild” (1–2 points), “moderate” (3–4) and “severe” (> 4 points) comorbidity. The second comorbidity index we used was the adult comorbidity evaluation-27 (ACE-27).^18^ The ACE-27 grades specific diseases and conditions into levels of comorbidity from grade 1 to grade 3. An overall level of comorbidity (“none”, “mild”, “moderate” or “severe”) is assigned based on the highest level of comorbidity. Only malignancies other than mCRPC were included in the final score of both comorbidity indices as its inclusion would have assigned a severe level of comorbidity to all patients. We retrieved relevant clinical information from electronic and hard copies of patients’ charts and assessed comorbidity by using the aa-CCI and ACE-27 coding protocols.

In this retrospective study the number of hospital admissions due to the toxicity of chemotherapy or deaths, which occurred during or 30 days after treatment discontinuation represented an estimate of tolerability of docetaxel. Dates of death were obtained from the national cancer registry.

The protocol of our study was reviewed and approved for clinical use by the Ethics and Study Protocol Assessment Committee at the Institute of Oncology Ljubljana. Informed consent was obtained from all patients prior to the treatment; however, for including in this retrospective study it was waived.

### Statistical analysis

Descriptive statistics were used to describe relevant characteristics of men at baseline. OS was calculated from the date of the first administration of docetaxel to death from any cause. Data were censored for patients who were alive at the cut-off date of March 13, 2013. OS was estimated using the Kaplan-Meier method. A Cox proportional hazard model was used to examine association between comorbidity and OS. Initial assessment was carried out in the univariable setting and subsequently for all significant (p < 0.1) variables in the multivariable setting. Comorbidity was analysed as both categorical and dichotomous variable (score 0 vs. ≥ 1). Discriminatory accuracy of the aa-CCI and ACE-27 in predicting death at 12 months or at any time during follow-up was tested by estimating the area under the receiver operating characteristic (ROC) curve (C-statistic). Association between comorbidity, RDI and tolerability was assessed by the Chi-square test. All tests were two-sided and a p-value of ≤ 0.05 was considered statistically significant. No adjustment for multiple analyses was performed.

## Results

### Study population

Our analysis included 208 men with mCRPC with median age of 69.9 years. Patients were treated with docetaxel between January 2005 and June 2012. Their baseline characteristics are presented in Table 1. At baseline, median PSA, haemoglobin (Hb) and alkaline phosphatase (ALP) were 217 ng/ml (78.2–595.1ng/ml), 124g/l (108–134g/L) and 2.9 μ kat/L (1.57–7.2 μ kat/L), respectively. Visceral metastases were present in 34 patients (16%) and 91 patients (44%) received opioid analgesia. Dq3w was administered to 199 patients (96%) with the remaining 9 patients (4%) having received weekly docetaxel. Median number of cycles of docetaxel was 8 (1–21). Twelve patients (6%) were re-challenged with docetaxel. Data for calculation of the RDI were available for 196 patients (94%), among these 159 (81%) received a RDI of ≥ 85%. After treatment with docetaxel 46 men (22%) received new agents (i.e. abiraterone acetate and/or cabazitaxel); 29 patients (14%) received abiraterone acetate alone, 5 patients (2%) cabazitaxel alone and 12 patients (6%) both agents.

### Association of comorbidity and tolerability

One hundred ninety-four men (93%) with mCRPC for whom information about both the dose intensity of therapy with docetaxel and comorbidity were available were included into the analysis of tolerability. Median RDI of docetaxel was > 90% in all subgroups of men with various level of comorbidity. However, when assessed by the aa-CCI, 34 men (27%) with moderate or severe comorbidity received RDI of less than 85% as compared to only 2 men (3%) with mild or no comorbidities (p < 0.001). When assessed by ACE-27, 17 men (24%) with moderate or severe comorbidity received RDI of less than 85% as compared to 19 men (15%) with mild or no comorbidity (p = 0.14) (Table 2).

Overall, 77 men (37%) were hospitalized due to the toxicity of docetaxel or died during treatment with docetaxel. Among those for whom information on dose intensity was available 66 men (34%) were hospitalized or died during treatment with docetaxel. In these men, 71 hospitalisations and 8 deaths occurred. When assessed by aa-CCI, 41 men (33%) with moderate or severe comorbidity as compared to 25 men (36%) with no or mild comorbidity were hospitalized or died during treatment (p = 0.63). When assessed by ACE-27, 24 men (34%) with moderate or severe comorbidity required hospitalisations or died during treatment as compared to 44 men (34%) with no or mild comorbidity (p = 0.84) (Table 3).

### Association of comorbidity and efficacy

After a median follow-up time of 14 months 133 men died. Median OS for the whole group was 19 months. For 98% of patients (N = 205) information on comorbidity was available. None, mild, moderate and severe comorbidity was present in 2%, 32%, 53% and 13% using aa-CCI and in 27%, 35%, 29% and 8% when assessed by ACE-27 (Table 4).

In univariable analysis, a higher level of comorbidity was not associated with worse OS (ACE-27: HR 0.96; [95% confidence interval (CI) 0.79–1.17], p = 0.69; aa-CCI HR 0.99; [CI 0.87–1.13], p = 0.93) when studied as a categorical variable (Table 5). Similarly, when analysed as dichotomous variable a higher level of comorbidity was not associated with worse OS (ACE-27: HR 0.75 [CI 0.51–1.08], p = 0.12; aa-CCI: HR 1.48 [CI 0.37–6.0], p = 0.58). Both indices were poor at discriminatory accuracy in predicting death at any time (C-statistics 0.45; p = 0.25 for aa-CCI and C-statistics 0.47; p = 0.44 for ACE-27) or death within 12 months of start of treatment with docetaxel (C-statistics 0.47; p = 0.49 for aa-CCI and C-statistics 0.47; p = 0.49 for ACE-27).

In multivariable analysis significant independent predictors of poor OS were presence of visceral metastases (HR 1.73; p = 0.02) and weekly docetaxel schedule (HR 6.04; p < 0.01), whereas high Hb level (HR 0.78; p < 0.01) and use of abiraterone and/or cabazitaxel (HR 0.36; p < 0.01) were independent favourable prognostic factors for OS (Table 5).

## Discussion

One of the reasons that a substantial proportion of men with mCRPC are never treated with docetaxel is fear of poor tolerability due to comorbid conditions.^10^ Life expectancy of men with mCRPC cancer is limited and therefore the impact of comorbidity on the outcome may be less relevant in this setting as compared to men with early prostate cancer. As patients enrolled into clinical trials usually do not have uncontrolled or severe comorbidities, population-based outcome studies may provide a more generalizable evaluation of the impact of comorbidity on the outcome and tolerability of medical intervention compared to post-hoc analyses of randomized trials.

In our cohort of men with mCRPC treated with docetaxel, a substantial proportion had moderate or severe comorbidity. In this cohort, a substantial dose reduction of docetaxel occurred more often in men with moderate or severe comorbidity as compared to those with no or mild comorbidity. At all comorbidity levels about one-third of men required hospitalization or died during treatment with docetaxel. A higher level of comorbidity was not associated with worse OS, irrespective of whether comorbidity was assessed by the aa-CCI or ACE-27 index. Both indices performed similarly poorly at discriminatory accuracy in predicting death. In concordance with our findings, investigators of the population-based outcome study in France, which enrolled elderly (≥ 75 years) men with mCRPC, did not find any association between comorbidity and outcome.^19^

To date several post-hoc analyses of randomized clinical trials, which evaluated the impact of comorbidity on the outcome in patients with advanced prostate cancer or other cancers, were reported. In a randomized phase II trial in which men with mCRPC were treated with docetaxel and prednisone with or without a bcl-2 antagonist AT-101, comorbidity assessed by the CCI did not predict OS, both as a categorical or continuous variable.^15^ In this study patients with acute or uncontrollable comorbidity were excluded and 53% had no other comorbidities. In contrast, in the post-hoc analysis of a large phase III randomized trial, which enrolled men with mCRPC receiving docetaxel and prednisone with or without bevacizumab, investigators found an association between the number of comorbidities at baseline and the risk of death.^14^ However, in that study comorbidity was not assessed by any comorbidity index. The lack of association between comorbidity assessed by the CCI and OS is also seen in other malignancies.^20^,^21^ Investigators in one of these studies cautioned that physicians must carefully discriminate between PS and comorbidity when assessing patients for therapy.^21^ According to the updated guidelines for the management of men with advanced prostate cancer published by the International Society of Geriatric Oncology (SIOG) comorbidity should be put into the context of patient’s general well-being and functional reserve.^22^

A higher level of comorbidity is a known risk factor for hospital admission in cancer patients.^23^,^24^ Irrespective of the level of comorbidity about one third of men required hospitalization or died during or shortly after treatment with docetaxel in our study. However, a higher proportion of men with moderate or severe comorbidity had a substantial dose reduction of docetaxel as compared to those with no or mild comorbidity (24–27% vs. 3–15%). Recent data show that patients with mCRPC treated with docetaxel in routine practice experience more toxicity as compared to those treated with docetaxel within clinical trials.^5^ Similarly, high admission rates during treatment with chemotherapy, as found in our study, were observed in older patients with other metastatic solid cancers.^25^,^26^ Accumulating evidence shows that anticancer therapies are less tolerable in patients treated in everyday clinical practise as compared to those treated within clinical trials.

Recently, new treatment options with favourable benefit-risk profile such as abiraterone acetate, enzalutamide and radium-223 became available for chemotherapy-naïve men with mCRPC.^27^–^29^ These agents substantially changed the management of mCRPC as treatment with docetaxel may now be deferred or even omitted in some patients with very advanced prostate cancer. However, we believe that docetaxel remains an important treatment option for mCRPC and therefore our findings may still be clinically relevant.

Our study has several limitations. First, our retrospective analysis is based on a relatively small cohort of men with mCRPC, who were all treated at a single institution. However, all patients with mCRPC in Slovenia who are candidates for treatment with docetaxel are referred to the Institute of Oncology Ljubljana. Therefore, our study is less prone to selection and referral biases which often plague traditional institutional retrospective studies. Second, when assessing comorbidity using aa-CCI and ACE-27 coding protocols, we retrieved relevant clinical information retrospectively from electronic and hard copies of patient’s charts, which may have limitations. An ideal set-up to study the association between comorbidity and outcome in men with mCRPC would be a prospective study design. Third, although serum PSA and ALP levels and pain are well established prognostic factors in men with mCRPC, they did not independently predict the outcome in our study. Similar larger study might lead into different conclusions and more generalizable results. Fourth, use of alternative comorbidity indexes might lead to different results and conclusions. We used aa-CCI and ACE-27, as these are convenient, widely used and validated tools. Fifth, the rate of hospitalization during treatment might be underestimated as admissions to community hospitals might not always be recorded in patients’ charts. Finally, use of abiraterone acetate and/or cabazitaxel after treatment with docetaxel in 22% of patients might impact the results. However, these new agents were not used more often in men with moderate or severe comorbidities as compared to men with no or minor comorbidities (Table 1).

In conclusion, the presence of comorbidity may not be associated with worse outcome in men with mCRPC, who are treated with docetaxel. Irrespective of the level of comorbidity a substantial proportion of men (about one third) required hospitalization or died during therapy with docetaxel. Dose reduction occurred more often in men with moderate or severe comorbidity. It is likely that, men with mCRPC who have substantial comorbidities may still benefit from reduced dose of docetaxel. Comorbid conditions should always be interpreted in the context of other relevant clinical characteristics when deciding about therapy with docetaxel in men with mCRPC.

## Figures and Tables

**TABLE 1.** Patients’ baseline characteristics

**Age (years)**	
Median	69.9
Range	45.7 – 84.8
**PSA (ng/mL)**	
Median	217
IQR	78.2 – 595.1
**Hb (g/L)**	
Median	124
IQR	108 – 134
**ALP (μ kat/L)**	
Median	2.9
IQR	1.57 – 7.20
**Docetaxel, n (%)**	
Dq3w schedule	199 (96)
Weekly schedule	9 (4)
Number of cycles, n	
Median	8
Range	1–21
IQR	5–10
Rechallenge, n (%)	12 (6)
RDI of docetaxel^*^, n (%)	
> = 95%	103 (52)
85–94%	56 (29)
75–84%	25 (13)
< 75%	12 (6)
**RDI of docetaxel, %**	
Median	95
IQR	87–99
**G-CSF support, n (%)**	
Pegfilgrastim	30 (15)
Filgrastim	15 (7)
Primary prophylaxis	20 (10)
Secondary prophylaxis	25 (12)
**Visceral metastasis, n (%)**	34 (16)
**Opioid analgesic, n (%)**	91 (44)
**New agents, n (%)**	
Abiraterone acetate	41 (20)
Cabazitaxel	17 (8)
Abiraterone acetate and/or cabazitaxel	46 (22)^†^

* Data available for 196 patients

† Among these, 53% and 29% had moderate or severe comorbidity when assessed by the aa-CCI and ACE-27

ALP = alkaline phosphatase; CI = confidence interval;

G-CSF = granulocyte colony stimulating factor; Hb = haemoglobin;

IQR = interquartile range; RDI = relative dose intensity;

PSA = prostate specific antigen

**TABLE 2.** Association of relative dose intensity of docetaxel and comorbidity

		**aa-CCI (n = 194)**				**ACE-27 (n = 194)**			
	
		None (n = 3)	Mild (n = 66)	Moderate (n = 101)	Severe (n = 24)	None (n = 54)	Mild (n = 69)	Moderate (n = 58)	Severe (n = 13)
RDI	<75% n (%)	0	0	10 (10%)	2 (8%)	3 (6%)	3 (4%)	5 (9%)	1 (8%)
	75–84% n (%)	0	2 (3%)	19 (18%)	3 (12%)	3 (6%)	10 (14%)	11 (19%)	0
	85–94% n (%)	1 (33%)	19 (29%)	28 (28%)	8 (33%)	13 (24%)	18 (26%)	19 (33%)	6 (46%)
	≥ 95 n (%)	2 (66%)	45 (68%)	44 (44%)	11 (46%)	35 (65%)	38 (55%)	23 (40%)	6 (46%)
Median RDI - %		96%	98%	93%	93%	98%	96%	91%	94%

aa-CCI = age-adjusted Charlson comorbidity index; ACE-27 = adult comorbidity evaluation 27; CI = confidence interval; RDI = relative dose intensity

**TABLE 3.** Association of comorbidity and tolerability during treatment with docetaxel

	**aa-CCI (n = 194)**				**ACE-27 (n = 194)**			
	
	None (n = 3)	Mild (n = 66)	Moderate (n = 101)	Severe (n = 24)	None (n = 54)	Mild (n = 69)	Moderate (n = 58)	Severe (n = 13)
Number of patients hospitalized or deceased during treatment, n	0	25 (38%)	34 (34%)	7 (29%)	20 (37%)	22 (32%)	18 (31%)	6 (46%)

aa-CCI = age-adjusted Charlson comorbidity index; ACE-27 = adult comorbidity evaluation; CI = confidence interval

**TABLE 4.** Comorbidity evaluation

**Comorbidity index**	**Level of comorbidity**			

	**None**	**Mild**	**Moderate**	**Severe**
aa-CCI, *N* (%)	3 (2%)	67 (32%)	108 (53%)	27 (13%)
ACE-27, *N* (%)	55 (27%)	73 (35%)	61 (29%)	16 (8%)

aa-CCI = age-adjusted Charlson comorbidity index; ACE-27 = adult comorbidity evaluation 27

**TABLE 5.** Association of comorbidity and overall survival

	**Univariable model** (HR 95% CI); p-value	**Multivariable model** (HR 95% CI); p-value
Age (for every year)	0.99 (0.97–1.01) p = 0.47	-
Log PSA (≥ *vs*. than median)	1.20 (1.07–1.35) p < 0.01	1.05 (0.94–1.18) p = 0.38
Hb (per 10 units)	0.79 (0.72–0.86) p < 0.01	0.78 (0.72–0.86) p < 0.01
ALP (≥ *vs*. < than median)	0.97 (0.87–1.10) p = 0.65	-
Docetaxel (Dqw *vs*. Dq3w)	6.39 (3.19–12.81) p < 0.01	6.04 (2.77–13.20) p < 0.01
Visceral metastases (yes *vs*. no)	1.68 (1.08–2.61) p = 0.02	1.73 (1.10–2.73) p = 0.02
Opioids (yes *vs*. no)	1.38 (0.65–2.92) P = 0.41	-
New agents (yes *vs*. no)	0.39 (0.19–0.80) p = 0.02	0.36 (0.17–0.74) p < 0.01
aa-CCI (per category)	0.99 (0.87–1.13) p = 0.93	-
ACE-27 (per category)	0.96 (0.79–1.17) p = 0.69	-

aa-CCI = age-adjusted Charlson comorbidity index; ACE-27 = adult comorbidity evaluation 27; Dqw = weekly docetaxel; Dq3w = 3-weekly docetaxel; HR = hazard ratio; CI = confidence interval
